# Rhinocerebral Mucormycosis: An Emerging Threat in the Era of COVID-19

**DOI:** 10.7759/cureus.28057

**Published:** 2022-08-16

**Authors:** Deoda Maassarani, Georges F Bassil, Micheal Nehme, Anis Nassar, George Ghanime, Ziad Sleiman

**Affiliations:** 1 Department of Plastic Surgery, Lebanese Hospital Geitaoui-University Medical Center, Beirut, LBN; 2 Division of Orthopedic Surgery, Department of Surgery, Lebanese University Faculty of Medicine, Beirut, LBN; 3 Division of Head and Neck Surgery, Department of Surgery, Lebanese University Faculty of Medicine, Beirut, LBN; 4 Department of Radiology, Lebanese University Faculty of Medicine, Beirut, LBN; 5 Division of Plastic and Reconstructive Surgery, Department of Surgery, Lebanese University Faculty of Medicine, Beirut, LBN

**Keywords:** rhinocerebral mucormycosis, sars-cov-2, complications, covid-19, rhizopus, mucormycosis, rhinocerebral

## Abstract

Mucormycosis is a rare but aggressive and fatal infection that is prevalent in immunocompromised patients. The variation in its clinical presentation and the lack of specificity are misleading and lead to a delay in the diagnosis and management. However, the era of coronavirus disease 2019 (COVID-19) is marked by the increasing emergence of Mucor infections, now identified as coronavirus-associated mucormycosis (CAM). Although many clinical forms exist, the most encountered in CAM is rhino-orbito-cerebral, as already reported in India.

We present a case of a 56-year-old male patient with uncontrolled diabetes mellitus and a history of recent SARS-CoV-2 infection treated with IV steroids, presenting for maxillary teeth pain and instability on day 16 of COVID-19 infection. Early diagnosis of CAM is crucial and will help decrease mortality in COVID-19 patients, especially those with comorbidities such as diabetes mellitus. Increasing cases of CAM should prompt clinicians to have a high index of suspicion for rhinocerebral mucormycosis, especially in patients with risk factors receiving steroid therapy.

In such patients, baseline glycosylated hemoglobin level and strict glycemic control by frequently measuring blood glucose levels and strictly adhering to insulin protocols would be rational but its efficacy in limiting the numbers of CAM in developing countries still needs to be confirmed.

## Introduction

Rhinocerebral mucormycosis (RCM) most commonly develops after inhalation of the spores into the paranasal sinuses [1]. The role of a healthy immune system is evident in the pathogenesis of this disease, given the rareness of these infections in humans. Data have proven that these infections would only occur in humans with underlying conditions compromising the immune system; the most common risk factors are diabetes mellitus (with or without ketoacidosis) and hematologic malignancies. Other risk factors have been identified including treatment with glucocorticoids [2], a cornerstone in the management of severe coronavirus disease 2019 (COVID-19) pulmonary infections.

At first, the clinical presentation could be misleading, mimicking the symptoms of acute sinusitis or facial cellulitis; commonly reported symptoms are fever, seen in 44% of cases, nasal congestion and purulent discharge, headache, facial pain, dental pain, and decreased vision [3]. The lack of specificity of these symptoms coupled with the rapid spread of the infection to the surrounding structures such as the palate, the orbits, and the brain is responsible for its bad prognosis at the time of diagnosis associated with high mortality rates. Therefore, a high index of suspicion is needed for the diagnosis based on risk factors, history of present illness, and laboratory, microscopic, and imaging findings.

While COVID-19 cases are still on the rise worldwide despite the emergence of multiple vaccination protocols, the number of reported mucormycosis cases is still increasing. The vast majority of these are originating in India where the incidence of Mucor infections was 80 times higher than in developing countries way before the COVID-19 pandemic [4].

We report a case of a rhino-orbito-cerebral Mucor infection occurring in a diabetic patient on day 16 of his COVID-19 infection after receiving steroid therapy for severe pulmonary involvement. Our patient was managed aggressively with intravenous (IV) amphotericin, IV Tazocin, and clindamycin, coupled with both endoscopic and open surgical debridement.

## Case presentation

A 56-year-old male patient came to the emergency department complaining of severe left-sided frontal headache and left facial pain mainly on the left maxillary and periorbital area, with associated photophobia. History goes back to a few days prior to presentation when he started complaining of left posterior maxillary teeth pain and instability to which he sought dental care and surgery with the extraction of the 26-27-28 teeth, coupled with per os (PO) Augmentin treatment. During surgery, necrotic bone was found extending from the teeth sockets to the maxillary alveolar processes up to the anterior maxillary wall. Therefore, the patient was transferred to our facility for investigations and proper management after his symptoms got worse within a few days. Medical history revealed COVID-19 pulmonary infection with documented positive reverse-transcriptase polymerase chain reaction (RT-PCR) for severe acute respiratory syndrome coronavirus 2 (SARS-CoV-2) 10 days prior to presentation when he got admitted to the intensive care unit for three days due to severe respiratory distress and desaturation, after which he was transferred to a regular floor for additional two days. He received IV Decadron during his stay and steroids were then continued at home. The patient started complaining of non-specific teeth and gingival pain while he was still in the ICU. The patient had been complaining of arthralgia, myalgia, and upper respiratory symptoms with fever and chills for two weeks before he was documented positive for COVID-19, thus when admitted to our facility, he had already been infected for roughly 20 days. Additionally, our patient was known to have hypertension (on candesartan 16 mg PO once a day (OD)), uncontrolled diabetes mellitus (not taking any of the prescribed medications) with glycosylated hemoglobin (HbA1c) of 12% measured upon his ICU admission, and peripheral neuropathy (pregabalin 75 mg PO OD).

**Figure 1:**
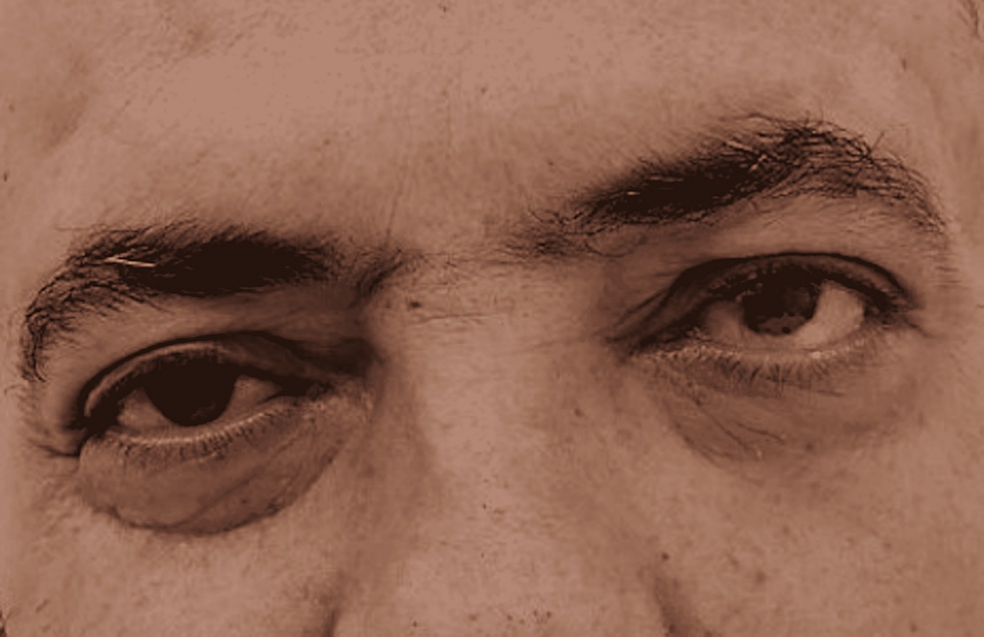
Pre-operative image showing vertical dystopia.

On extraoral examination, swelling was noted over the left midface involving the malar region and the periorbital tissues from the medial to the lateral canthi. Left ophthalmoplegia with dystopia (Figure 1) was noted associated with paresis of the sixth cranial nerve, limiting lateral deviation of the left eye. Cranial nerves examination showed no visual impairment, paresis of the frontal, zygomatic, and buccal branches of the facial nerve limiting the function of facial mimetic muscles, and paresthesia in the territory of the maxillary branch of the trigeminal nerve was also noted. Upon palpation, soft tenderness was noted over the swollen areas with no palpable isolated mass and no palpable cervical lymph nodes. Intra-oral examination showed extracted 26-27-28 teeth with swelling of the sutured gingiva; no gross secretions were noticed. Mobility of the 23-24-25 teeth was noted with additional pain over this territory extending to the right maxillary area but with no visible gross abnormality. Intranasal examination showed black discoloration of the left inferior turbinate and white discoloration of the head of the middle turbinate.

**Figure 2:**
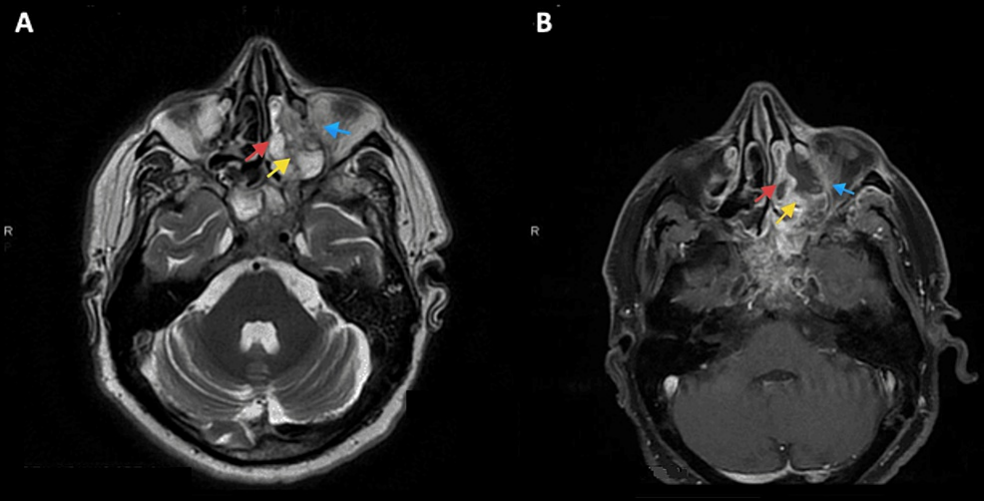
(A) Axial T2-sequence showing thickened hypointense lesion of the medial wall of the maxillary sinus (yellow arrows), orbital floor (blue arrows), and middle turbinate (red arrows). (B) Axial T2 fat-saturated image showing no enhancement of the necrotic medial wall of the maxillary sinus, orbital floor, and middle turbinate.

**Figure 3:**
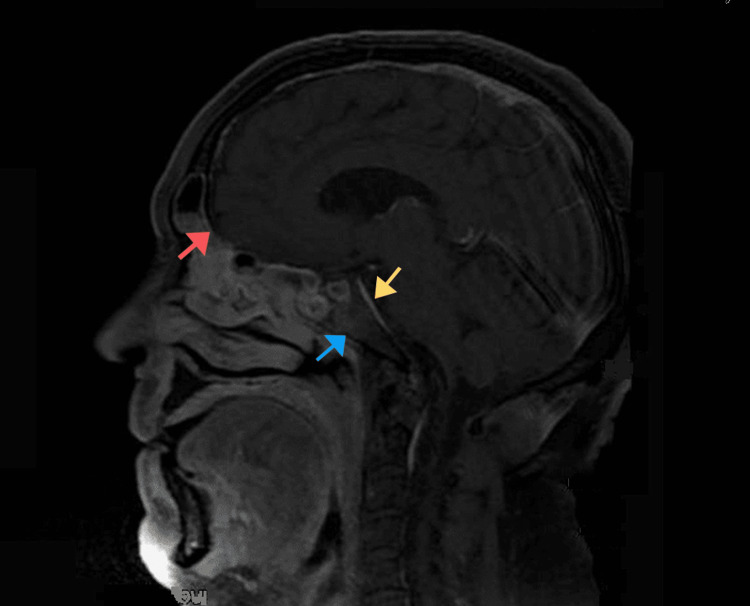
Sagittal T1 fat-saturated image showing abnormal enhancement of the anterior surface of the clivus (blue arrow), the dura (red arrow), and the prepontine cistern (yellow arrow).

Based on historical and clinical findings, a preliminary diagnosis of mucormycosis was established. The patient was admitted and a set of labs along with a CT scan of the face with IV contrast were ordered. As expected, labs showed an inflammatory pattern with elevated white count and C-reactive protein (CRP). CT with IV contrast showed heterogenous bone density at the left alveolar process of the maxilla associated with surrounding air bubbles and extending to the maxillary zygomatic process; furthermore, complete opacification of the left maxillary, sphenoid, ethmoid sinus, and partially of the left frontal sinus was noted, mimicking pansinusitis. MRI with IV gadolinium (Figure 2) showed extensive necrosis of the maxillary bone mainly at the medial and anterior walls, zygomatic arch, ethmoidal air cells, orbital floor (in the extraconal region), and middle turbinate. At the posterior aspect of the orbital floor, the inflammation was inseparable from the inferior rectus muscle. The edema also involved the infraorbital canal and foramen, correlating with clinical findings. The disease was also shown to have an intracranial extension (Figure 3), with an abnormal enhancement of the anterior surface of the clivus, the dura, and the prepontine cistern.

**Figure 4:**
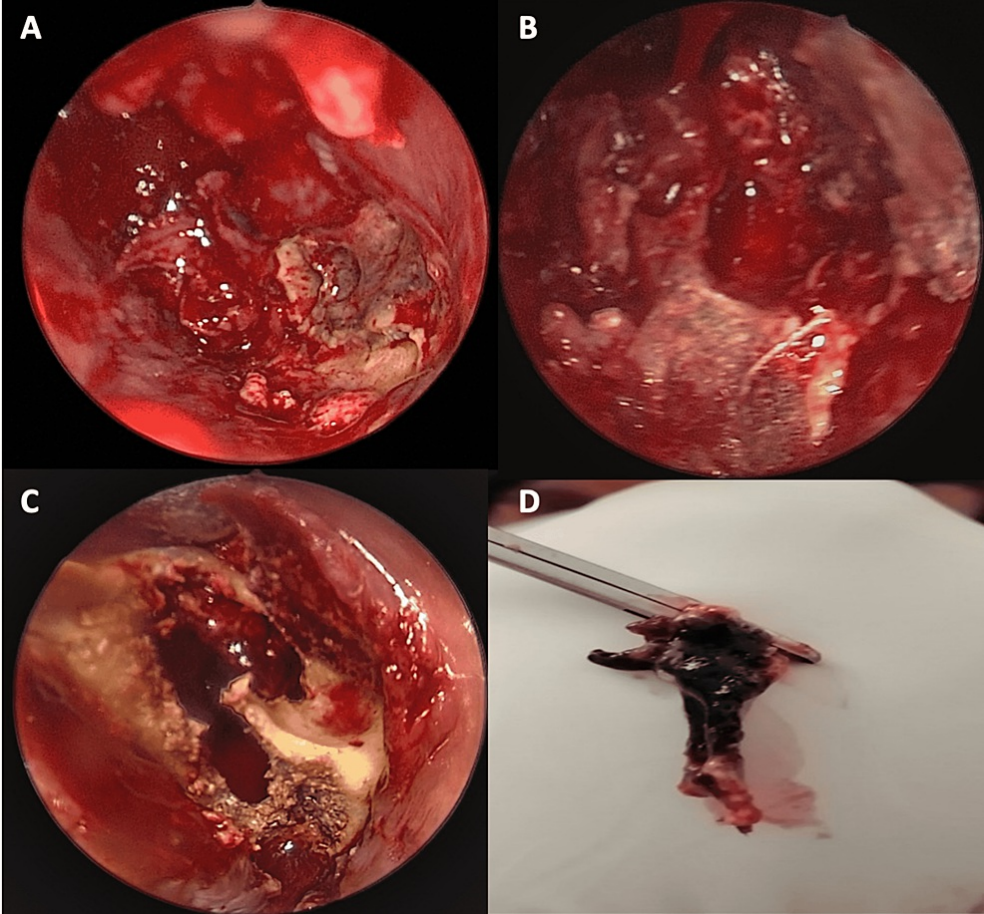
(A) Gross involvement of the middle and inferior turbinate. (B) Status post removal of the middle turbinate and debridement of the necrotic posterior maxillary wall. (C) Open maxillectomy showing necrotic alveolar bone. (D) Resected necrotic inferior turbinate.

**Figure 5:**
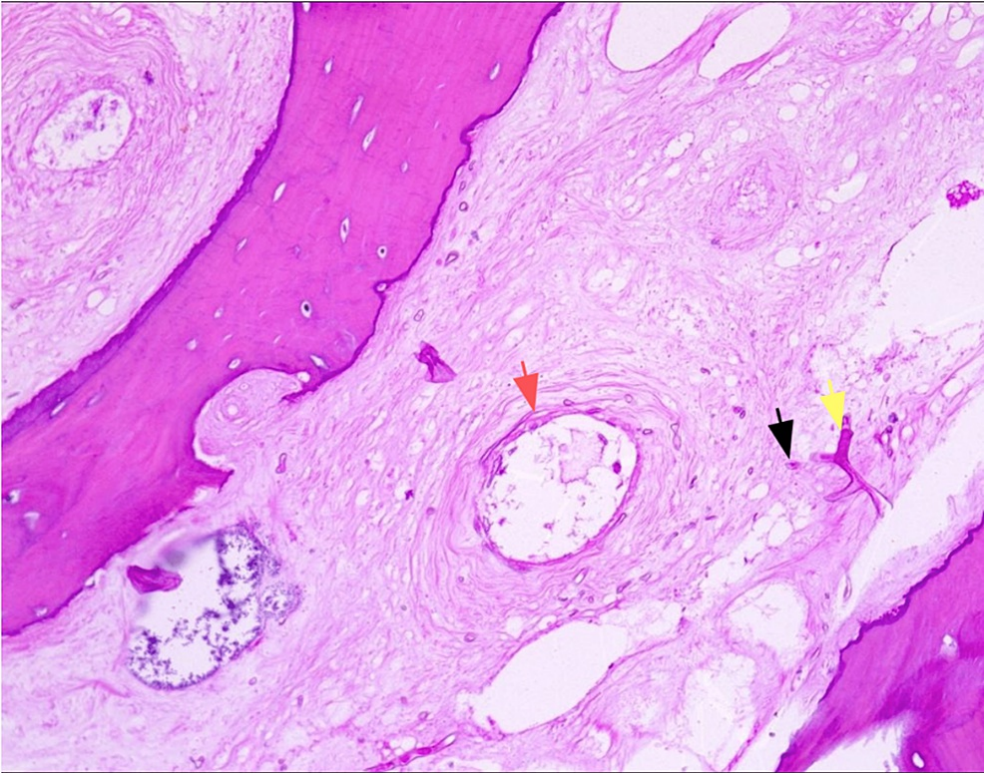
Microscopic image of the specimen taken intraoperatively showing the presence of Mucor spores (black arrow) and mycelium (yellow arrow) with evidence of angioinvasion (red arrow).

The patient was started immediately on amphotericin B at a dose of 5 mg/kg/day associated with Tazocin and clindamycin. Urgent surgical debridement was performed after starting antifungal therapy: an endonasal approach with a 30-degree scope was used to debride necrotic tissue. We noted gross involvement of the middle and inferior turbinates (Figure 4A) with necrosis extending to the inferior meatus. We removed the middle turbinate entirely until its skull base attachment with debridement of the necrotic posterior maxillary wall (Figure 4B), proceeded with anterior and posterior ethmoidectomy, the opening of the frontal recess, followed by large sphenoidotomy. Inflammatory tissue was removed from the sphenoid sinus and no necrotic tissue was visualized in the clivus. The inferior turbinate was also resected along with the necrotic medial wall of the maxillary sinus. The procedure was then converted to an open maxillectomy due to the involvement of the hard palate; an intra-buccal approach through the left upper buccal sulcus was used to debride necrotic alveolar bone (Figure 4C) and to access the maxillary sinus. Burring of the anterior wall was performed with the removal of necrotic bone, and the sinus cavity was accessed, debrided, and thoroughly cleaned. The posterior maxillary wall was removed and the sphenoid bone was visualized to be grossly normal. Samples of tissues were sent for pathology (Figure 4D), which confirmed the diagnosis by describing typical ribbon-like, minimally septate hyphae invading surrounding blood vessels (Figure 5). Also, cultures from the specimen were positive for mucormycosis. A polymerase chain reaction test on the specimen was not performed.

**Figure 6:**
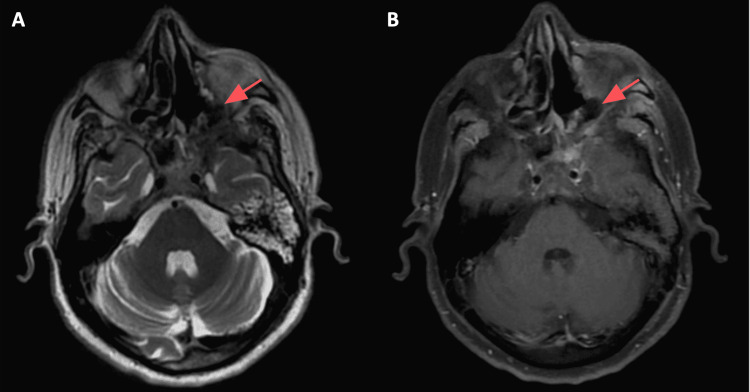
Postoperative MRI images: axial T2 (A) and axial T1 (B) with fat saturation showing resolution of the mucormycosis infection (red arrows showing the site of the previous mucormycosis infection).

**Figure 7:**
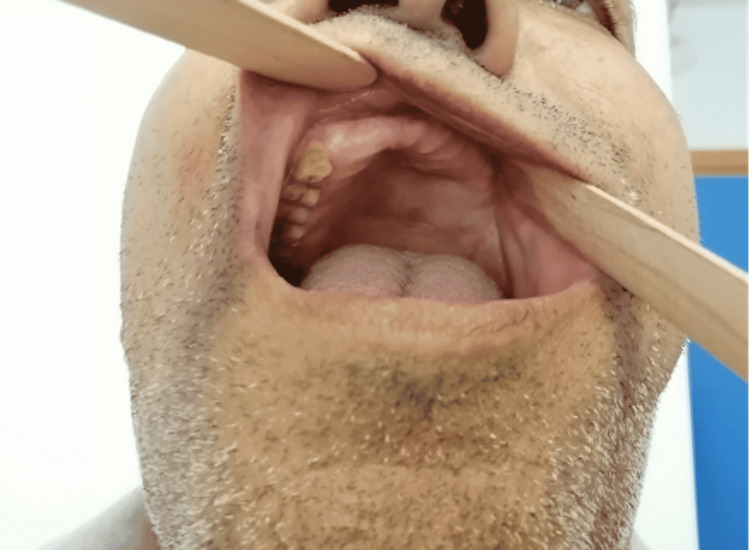
Intra-oral exam showing healthy gingiva and palate postoperatively.

On day seven postoperatively, a second endoscopic nasal evaluation was performed to remove residual necrotic tissue. Our patient took a two-month course of amphotericin B, which was interrupted on three occasions due to a disturbance of liver function tests. By the end of the second month, he was switched to voriconazole, which was continued PO at home for additional two months. On follow-up, imaging showed resolution of infection (Figure 6), and clinically, intra-oral examination showed healthy tissues (Figure 7) with normal gingiva and palate with no apparent fistulas.

## Discussion

Like many other viral infections, the COVID-19 infection caused by the SARS-CoV-2 has been associated with either bacterial or fungal infections. Co-infections have been developing either as hospital-acquired infections or based on pre-existing comorbidities such as diabetes mellitus or chronic pulmonary diseases [5]. Multiple cases of severe COVID-19 infections have been reportedly complicated by pulmonary aspergillosis, giving rise to a new disease entity called COVID-19-associated pulmonary aspergillosis (CAPA) [2]. Furthermore, mucormycosis, another fungal infection, seems to be a rising concern for COVID-19 patients, especially in those who have risk factors shared by both disease entities. The common scenario is that of a patient with uncontrolled diabetes mellitus receiving high doses of parenteral corticosteroids for the management of SARS-CoV-2 pulmonary infection [2]. This arising entity, with actual cases being reported in India, is referred to as coronavirus-associated mucormycosis (CAM) [6].

In fact, the fungi responsible for the disease belong to the genera of *Mucorales* (a subtype of *Zygomycetes*), which are responsible for most fungal human infections. These pathogens reproduce by forming zygospores, ubiquitously contaminating fruits, soil, and feces, thus causing infection whenever an immunosuppressed territory is provided [7]. The spores are inhaled and are given entry to the paranasal sinuses and maxillofacial area commonly through mucosal ulceration or an intraoral extraction wound [7,8]. It is in the alveoli or the nasal turbinate that these angioinvasive fungi would start damaging tissues, making blood vessel thrombosis the hallmark of this infection. By causing vascular thrombosis, healthy tissues become rapidly necrotic, and an acidic anaerobic environment is established, allowing for further growth of the fungi [9] due to their ketone reductase enzyme [10]. Most commonly, patients present initially with facial and periorbital swelling, only rarely with tooth pain, as seen in our case presentation [11].

Data have proven that these infections would only occur in humans with underlying conditions compromising the immune system. A series of case reports collected between 1940 and 2003 found that the most common risk factor for the invasive disease was diabetes mellitus, followed by hematologic malignancies and various solid organ and bone marrow transplantations [12]. This was followed by another study in France in the early 2000s, showing that hematologic malignancies were the leading risk factor for mucormycosis (50%), diabetes mellitus being found in 23% of cases [13]. Studies found that COVID-19 patients are at increased risk to develop acute diabetes mellitus and diabetic ketoacidosis (DKA) due to direct damage of the pancreatic islet cells by the SARS-CoV-2 particles [14] as well as the increasing insulin resistance due to the systemic inflammatory response mediated by the pro-inflammatory cytokines. Additionally, treatment guidelines concerning severe cases of COVID-19 infection recommend the use of corticosteroids either in the form of methylprednisolone or dexamethasone (6 mg per day for a maximum of 10 days in patients who are ventilated or requiring supplemental oxygen therapy as recommended by the National Institute of Health), which in turn disrupts the glycemic homeostasis predisposing to opportunistic mycotic infections including mucormycosis.

RCM is an invasive and rapidly progressive infection that results in irreversible damage to the surrounding tissues. That is why early diagnosis is of primordial importance. The clinical presentation of patients with RCM is variable and not specific. The patient may have symptoms of headache, nausea, fever, nasal congestion, facial numbness or weakness, periorbital cellulitis with retro-orbital pain and diplopia, amaurosis, and blurred vision that may progress to full blindness [15]. Treatment should not be delayed until confirmation of diagnosis. Necrotic patches at the palate or the intranasal region are highly suggestive of the disease, but they occur only in 50% of cases [16]. Imaging modalities (CT scan and MRI) may help determine the extent of the disease, the response to treatment, and the surgical approach if needed [17]. Histopathological examination and culture of specimen taken during surgical debridement will confirm the diagnosis [18].

Keys to successful management of mucormycosis include, in addition to early diagnosis and control of risk factors, antifungal therapy and surgical debridement when possible. Studies have shown that appropriate antifungal therapy, with polyenes being the first line agents, started within five days of diagnosis is associated with the highest survival rate (83% vs. 49% when initiated after six days) in patients with hematological malignancies [19]. Management of ketoacidosis and maintaining glycemic control using insulin therapy with sodium bicarbonate has proven to be necessary for the armamentarium of eradicating mucormycosis [20]. Amphotericin B, as recommended by the European Conference on Infections in Leukaemia (ECIL-6) in 2016, is the first-line agent for mucormycosis at a dose of 5 mg/kg/day and up to 10 mg/kg/day whenever the central nervous system is involved.

Although a 2008 study proved the benefits of combining amphotericin B and caspofungin in the management of RCM with improved survival rates [21], combination therapy remains controversial with other studies failing to support its efficacy [22].

Aggressive surgical debridement remains the cornerstone of mucormycosis treatment, especially rhino-orbito-cerebral mucormycosis without CNS involvement, optimizing outcomes; however, surgery showed no benefits in improving survival in patients with rhino-orbito-cerebral mucormycosis with CNS involvement [23]. The surgeon must not only remove macroscopically necrotic tissue but also surrounding probably infected healthy-looking tissues to hinder the progression of the disease [20]. However, a retrospective study evaluated the “aggressively conservative” surgical approach to mucormycosis during which serial tissue samples are sent for frozen section analysis to delineate the exact diseased margins and save as much tissue as possible [21]. Thus, the surgical approach when it comes to such a rapid and exceedingly destructive pathology remains a matter of controversy.

Finally, the SARS-CoV-2, regardless of the patient’s comorbid conditions, entails specific features that encourage opportunistic fungal infections: reducing the number of CD4+ and CD8+ T lymphocytes and altering the innate immunity would permit fungal proliferation in already diseased and damaged pulmonary alveoli [24]. Our patient developed symptoms suggestive of rhino-orbito-cerebral mucormycosis after he had been infected with COVID-19 for several days and had been on steroids for three days, which is a very short period compared to other newly reported cases in which steroid therapy was given for an average of seven days [25]. This clearly emphasizes the synergistic role of both the SARS-CoV-2 infection and the steroid treatment given to patients with severe pulmonary COVID-19, in addition to patient-specific risk factors such as uncontrolled diabetes mellitus, as in our case.

## Conclusions

The emergence of mucormycosis infections in COVID-19 patients during this pandemic should warrant a high index of suspicion to allow early intervention and treatment, thus avoiding additional mortality and morbidity. Additionally, it seems mandatory that all COVID-19 patients, especially those with diabetes mellitus receiving steroid treatment, undergo strict glycemic control during and after their hospitalization for the viral illness. HbA1c level should be ordered upon admission before steroids are started, and blood sugar levels should be monitored routinely once therapy is initiated with narrow and strict insulin protocols.
